# Exposure to Environmental Pesticides and the Risk of Autism Spectrum Disorders: A Population-Based Case-Control Study

**DOI:** 10.3390/medicina60030479

**Published:** 2024-03-14

**Authors:** Pablo Román, Cristofer Ruiz-González, Lola Rueda-Ruzafa, Diana Cardona, Mar Requena, Raquel Alarcón

**Affiliations:** 1 Department of Nursing, Physiotherapy and Medicine, Faculty of Health Sciences, University of Almeria, Carretera Sacramento s/n, La Cañada, 04120 Almeria, Spaindcardona@ual.es (D.C.); mrm047@ual.es (M.R.); ralarcon@ual.es (R.A.); 2 Research Group CTS-1114 Health Sciences, University of Almeria, 04120 Almeria, Spain; cristofer4@outlook.es; 3 Health Research Center, University of Almeria, Carretera Sacramento s/n, La Cañada, 04120 Almeria, Spain; 4 Torrecárdenas University Hospital, Calle Hermandad de Donantes de Sangre, s/n, 04009 Almeria, Spain

**Keywords:** pesticides, autism spectrum disorder, environmental exposure, prevalence, case-control studies

## Abstract

*Background and Objectives:* Autism spectrum disorder (ASD) is a neurodevelopmental condition characterized by challenges in communication, social interactions, and repetitive behaviors. Although the factors that influence the development of this condition are unknown, certain chemical compounds such as pesticides have been proposed as possible contributors. Due to the lack of an established causal link between pesticide exposure and ASD, this study aimed to evaluate this potential association. *Materials and Methods*: A case-control study was carried out to ascertain the prevalence and risk associated with ASD in relation to pesticide exposure over a 21-year study period (2000–2021). *Results*: We included 2821 individuals diagnosed with ASD residing in areas of both high and low pesticide exposure in southern Spain. There was a rise in the ASD prevalence rate in regions with elevated pesticide use when compared to regions with low use [odds ratio (OR): 1.34, 95% confidence interval (CI), (1.24–1.44)]. Notably, men had the highest likelihood, with an OR: 1.42, 95% CI, (1.30–1.55). Furthermore, after performing multiple binary logistic regression adjusted for age, sex, and geographical area, males exhibited a higher likelihood compared to females [OR: 2.41, 95% CI, (2.21–2.62)]. *Conclusions*: Overall, this research suggests a connection between heightened environmental pesticide exposure due to increased agricultural use and autism.

## 1. Introduction

Autism spectrum disorders (ASD) encompass a range of persistent deficiencies that hinder an individual and their development, with lasting consequences that change their cognitive processes, behavior, and interactions with the environment throughout their lifetime [1]. Although individuals with ASD share certain characteristics, the condition manifests in a variety of behaviors and expressions. These typically include communication difficulties, impairments in social interaction, and repetitive patterns of behavior [1,2]. At an epidemiological level, several studies conducted in different countries have reported a rise in the prevalence of ASD in the last 20 years [3,4]. This apparent increase in prevalence can be attributed to earlier recognition and improved access to health care and diagnostic criteria [5].

Although much remains unclear about the neurobiological basis of autism, histopathological and biochemical findings indicate that developmental changes occur in the structure and function of neurons in the amygdala, hippocampus, and frontal and temporal cortices [6]. These changes are associated with prominent cellular disorganization and alterations in neuronal size [7]. Furthermore, research indicates that dysfunction in both glutamatergic excitatory, and GABAergic inhibitory systems may contribute to the pathogenesis of ASD [8,9]. The disorder is believed to be influenced by a myriad of factors, including genetic predisposition, advanced parental age, complications during pregnancy and childbirth, prenatal infections, and exposure to chemical substances [10,11,12]. 

In recent years, there has been a growing focus on investigating the role of environmental pollutants in ASD etiology [13,14]. Studies have revealed that maternal exposure to air pollution containing nitric oxide or fine particulate matter, pesticides, or solvents elevates the risk of ASD development in offspring [14,15,16]. In this regard, prenatal exposure has been extensively investigated, showing that exposure to pesticides during pregnancy or via breast milk reveals associations with diminished cognitive function, verbal comprehension, social–emotional scores, memory, and motor alterations in children [17,18,19,20]. However, other studies have not identified significant neurological or psychomotor effects of organochlorine compounds in prenatally exposed children [21]. Similarly, research focusing on organophosphates, pyrethroids, or carbamate compounds has also yielded contradictory results [22,23,24,25]. Certain researchers have highlighted the potential adverse effects of pesticide combinations, suggesting that such mixtures could lead to compromised verbal comprehension, reduced motor speed, and impaired short-term memory, even potentially increasing the risk of autism after both pre and postnatal exposure [26,27,28,29].

Various experimental studies have explored the impact of pesticides on rodents. In animal models, exposure to chlorpyrifos during prenatal stages has demonstrated effects on sociability and is linked to behaviors resembling those observed in animals genetically susceptible to ASD. It has also been linked to delayed motor skill development characterized by the continuation of immature movement patterns [30,31]. Additionally, research has evidenced that exposure to glyphosate during crucial developmental periods can elicit neurobiological modifications and behavioral shifts akin to those observed in autism [32]. This highlights a potential correlation between glyphosate exposure and the emergence of ASD-like traits during critical stages of development. 

The underlying mechanism of these pesticides could involve inducing the generation of reactive oxygen species in areas involved in behavior, such as the prefrontal cortex and hippocampus [33,34]. Consequently, the landscape of research underscores the intricate nature of elucidating the precise impact of pesticide exposure on neurodevelopmental outcomes. It highlights the necessity for further comprehensive investigations to unravel the complex interplay between various pesticide compounds and their potential consequences on neurodevelopment and neurobehavioral disorders such as autism, all while maintaining a considered approach to the subject matter.

Recently, there has been a growing emphasis on investigating the link between autism and the gut–brain axis (GBA). The GBA is a bidirectional communication system between the intestine and the brain, where the intestinal microbiota consisting of bacteria, viruses, archaea, yeasts, and protists in the mucosa plays a crucial role [35]. In this regard, a significant incidence of gastrointestinal disorders has been identified among children with ASD compared to children with typical development [36]. Furthermore, there have been indications that individuals with autism who manifest gastrointestinal symptoms experience an intensification of repetitive behaviors and stereotypies [37]. 

It is important to note at this juncture the disruptive effects of several pesticides, including organophosphates and neonicotinoids, on the intestinal epithelium and composition of the intestinal microbiota [38,39]. Regarding glyphosate, it seems obvious how it can impact the microbiota since it inhibits the enzyme 5-enolpyruvylshikimate-3-phosphate synthase (EPSP synthase) in plants, hampering the production of aromatic amino acids [40]. This enzyme, essential for cellular survival in some organisms, is also found in bacteria, archaea, and fungi but is absent in humans, meaning that glyphosate could disrupt the balance of intestinal microorganisms and promote the growth of pathogens [41,42]. Meanwhile, diquat and imidacloprid, among other pesticides, have the potential to disturb the integrity of tight junctions in the epithelium, causing a disruption in cell cohesion and compromising the permeability of the intestinal barrier [43,44,45].

On the other hand, it is worth noting the widespread use of pesticides globally, particularly in the agricultural and crop protection industry. This practice is aimed at mitigating the risks posed by pests, diseases, and weeds, with the goal of preserving crop productivity and quality [46]. Agriculture plays a pivotal role in the Spanish economy, with the country recognized as a leading producer of fruits, vegetables, olive oil, wine, and various other agricultural commodities in Europe. However, despite its benefits, the application of pesticides raises significant concerns, including issues such as water and soil pollution, pest resistance to these chemicals, and the potential implications for human health [47]. Spanish agricultural practices heavily rely on the use of herbicides like glyphosate and pendimethalin, fungicides such as mancozeb and copper oxychloride, and insecticides like paraffin oil, all of which are considered essential phytosanitary measures [48]. These substances are regulated and undergo rigorous testing to evaluate their efficacy and safety, and policymakers consistently reassess them to ensure the protection of the populace.

The substantial variability in findings, combined with the intricate nature of autism as a multifaceted disorder influenced by numerous factors, highlights the imperative for expanded research endeavors aimed at elucidating the potential association and clinical relevance of the link between pesticides and autism. Thus, there is a pressing need for further investigation to understand in depth the intricacies of this relationship and its potential implications in the clinical setting. The primary aim of the current study is to assess whether environmental exposure to pesticides among individuals residing in proximity to regions characterized by intense agricultural activity correlates with an elevated risk of developing ASD over the course of a comprehensive 21-year timeframe.

## 2. Materials and Methods

### 2.1. Design

A case-control study was conducted in the autonomous community of Andalusia, located in southern Spain, following the reporting guidelines of the Strengthening the Reporting of Observational Studies in Epidemiology (STROBE) criteria [49]. This study aimed to investigate the potential association between exposure to pesticides and ASD within the population.

### 2.2. Criteria for Selecting Study Areas and Categorizing Pesticide Exposure

With a sample size of 52,393 individuals, the health districts in Andalusia were categorized according to agronomic criteria provided by the Andalusian Council of Agriculture, in particular tons of pesticide used, which was considered as a surrogate for pesticide exposure, and land surface devoted to intensive agriculture in plastic-covered greenhouses. In contrast to open field crops, the micro-climate conditions within these greenhouses are favorable to the development of pests and diseases such that farmers rely on pesticides to protect their crops. The high pesticide usage category encompassed four distinct health districts: West of Almería, Almería Center, South of Granada, and Huelva Litoral. Conversely, the remaining health districts, including Axarquía (Málaga), the coastal region of Jerez (Cádiz), East Almeria, Northeast Jaen, North Córdoba, and North Seville, were classified as low pesticide usage districts [50].

According to the report released by the Consejería de Agricultura Pesca y Desarrollo Rural from Andalusia (Spain) in 2017, data from the 2016–2017 crop season revealed that a substantial portion of pesticides, totaling 14,002 tons (91.4%), were utilized in regions classified as having high pesticide usage. These high-usage regions corresponded to 93.2% of the total surface area of plastic-covered greenhouses in Andalusia, which serve as cultivation sites for various fruits and vegetables. In contrast, areas characterized by low pesticide usage accounted for a mere 8.6% of the total pesticide consumption, amounting to 1306 tons. These regions occupied only 6.8% of the overall greenhouse surface area in Andalusia. The significant disparity in pesticide usage between high and low-usage regions underscores the pronounced variation in agricultural practices across different areas within the region [51].

During the study period, insecticides used in crops grown in plastic greenhouses primarily consisted of various chemical classes. These included organophosphates such as chlorpyrifos, chlorpyrifosmethyl, dimethoate, and pyrimifos-methyl, as well as N-methylcarbamates like methomyl and oxamyl. Macrocyclic lactones such as abamectin and spinosad, neonicotinoids including imidacloprid and acetamiprid, pyrethroids like cypermethrin and deltamethrin, and other substances like amitraz, formetanate, indoxacarb, azadirachtin, spiromesifen, *Bacillus thuringiensis*, and endosulfan were documented. In addition, the fungicides commonly used for plastic greenhouse crops included (di)thiocarbamates like zineb, mancozeb, maneb, and thiram; conazoles such as tebuconazole, triadimenol, myclobutanil, and prochloraz; dicarboximides like procymidone, iprodione, and vinclozolin; anilino-pyrimidines including cyprodinil, mepanipyrim, and pyrimethanil; copper salts like copper oxychloride; and other substances such as cymoxanil, metalaxyl, fosetyl, thiophanate methyl, fluopicolide, chlorthalonil, propamocarb, dimethomorph, and azoxystrobin. The most frequently used herbicides in the study areas were bipyridyls like paraquat and diquat; organophosphonates such as glyphosate and glufosinate; chlorotriazines like atrazine, simazine, terbuthylazine, and cyanazine; and phenylureas like isoproturon, linuron, diuron, and monuron [52].

### 2.3. Study Population and ASD

The study involved a population of 52,393 individuals from both areas. During the years 2000 to 2021, 2821 ASD cases were diagnosed. To form the control group, individuals of matching age and gender were selected from the general population residing in the same study regions as the cases. The Andalusian Public Health Service’s Basic Minimum Data Set (BMDS), which collects data on patient discharges from public hospitals, was utilized to identify both cases and controls. The BMDS records coded clinical information such as primary and secondary diagnoses, age, gender, and place of residence, and its accuracy is dependent on the quality of discharge reports and the comprehensiveness of coding for hospital discharges.

Andalusian patients without developmental disorders were included in the control group and were selected using a stratified random sampling method with health districts as the strata. The controls were matched to the cases based on age and gender, and a random sample of patients was selected from each stratum. The number of controls selected from each stratum was proportional to the number of cases diagnosed in that stratum during the study period. The control group primarily consisted of individuals residing in municipalities located more than 2 km away from the selected greenhouses for this study.

During the study, the identification of ASD cases was based on the criteria outlined by the World Health Organization in the ninth and tenth editions of the International Classification of Diseases (ICD-9 and ICD-10), respectively. Specifically, pervasive developmental disorders cases (PDD) were diagnosed using the ICD-9 code 299, autistic disorder 299.0 (being current or active status, code 299.00, and residual status, 299.01), childhood disintegrative disorder 299.1 (being current or active status, code 299.10, and residual status 299.11), other specified PDD 299.8 (being current or active status, code 299.80, and residual status, 299.81) and PDD not otherwise specified 299.9 (being current or active status, code 299.90, and residual status, 299.91). The ICD-10 codes used were F84 for pervasive developmental disorders, F84.0 for autistic disorder, F84.2 for Rett syndrome, F84.3 for another type of disintegrative disorder, F84.5 for Asperger syndrome, F84.8 for other pervasive developmental disorders, F84.9 for pervasive developmental disorders not otherwise specified, F88 for other disorders of psychological development, and F89 for unspecified developmental disorders of psychological development.

### 2.4. Statistical Analysis

Categorical variables were analyzed using frequencies and percentages, while means and standard deviations were utilized to assess quantitative variables. The prevalence proportion and risk of ASD in areas with high and low pesticide use were calculated, and the Chi-square test was employed to determine statistical significance. Additionally, odds ratios (OR) and 95% confidence intervals (CI) were calculated. The Kolmogorov–Smirnov normality test was utilized to compare age differences between the study areas, followed by the Mann–Whitney test. Multiple binary logistic regression was performed to assess the risk of developing ASD adjusted for age, gender, and areas of high versus low pesticide use as an indicator of exposure, as these factors were considered to impact the statistical models. A significance level of *p* < 0.05 was set for statistical significance. The data were analyzed using SPSS (Version 26.0, Armonk, NY, USA) statistical software.

### 2.5. Ethical Considerations

This study obtained ethical approval from the Provincial Research Ethics Committee of Almeria (UALMICROBIOTA21, 28 July 2021) and adhered to the ethical principles of the Helsinki Declaration and its subsequent revisions. To safeguard confidentiality and privacy, all data collected during the study was handled in accordance with current data protection laws, including Organic Law 3/2018 on the Protection of Personal Data and the Guarantee of Digital Rights; R.D. 994/99, Art. 5 of the General Data Protection Regulation; and the provisions of Law 41/2002, which regulate Patient Autonomy and the Rights and Obligations Regarding Clinical Information and Documentation.

## 3. Results

The study population consisted of 52,393 residents from the study areas, 2821 of whom were diagnosed with ASD between January 2000 and December 2021. Of the total individuals with ASD, 1536 people lived in areas of high pesticide use and 1285 people lived in areas of low pesticide use.

The mean age of individuals diagnosed with ASD in areas of high pesticide use was 4.96 (3.34) years, while in areas of low pesticide use, the mean age was 5.15 (3.05) years. No statistically significant differences were observed (Table 1). The mean age of the control population in areas of high pesticide use was very similar to that in areas of low pesticide use, 4.90 (2.17) years vs. 5.01 (2.56) years (*p* > 0.05).

The prevalence rates of ASD per 100 inhabitants were significantly higher in geographic areas with higher pesticide use compared to those in areas of lower pesticide use (Table 2). When the data were stratified by sex, these prevalence rates were significantly higher in both girls and boys in areas of high pesticide use.

Table 2 also displays the likelihood of having ASD expressed as the odds ratio (OR) for areas with high pesticide use in relation to those with low use. A significant increase in the odds of ASD was found in areas with high pesticide use compared to those with low use (OR: 1.34). The highest odds were observed for men, with an OR of 1.42.

Table 3 displays the multiple logistic regression analysis of ASD adjusted for sex and environmental exposure to pesticides. Individuals living in areas with high pesticide use showed a higher likelihood of developing ASD (OR: 1.52). Men also showed a significantly higher likelihood than women (OR: 2.41). In all cases, the results were statistically significant.

## 4. Discussion

The aim of this research was to investigate whether individuals living in proximity to areas with intensive farming, where they are frequently exposed to pesticides, exhibit a higher risk of ASD. This study involved a substantial population sample and comprehensive analysis of ASD in relation to pesticide exposure. While the mean age of ASD diagnosis did not significantly differ between high and low pesticide use areas, the prevalence rates of ASD were notably higher in regions with increased pesticide usage. Furthermore, the odds of developing ASD were significantly elevated in high pesticide use areas, particularly for males, as suggest by the multiple logistic regression analysis. 

Pesticide usage is prevalent in Spain, primarily by the agricultural industry, resulting in agricultural workers facing the highest risk of occupational exposure. Andalusia has the largest number of agricultural workers compared to other regions in Spain, with approximately 255,500 farmers in 2019, comprising 8.2% of active workers, twice the national average (4.0%) [53]. However, the general population may be impacted by environmental and food contamination as well, depending on their proximity to areas with high pesticide use [54,55,56]. All of the above justifies the realization of this study with the aim of elucidating the relationship between pesticide exposure and the prevalence of ASD. The results of this study suggest a connection between pesticides and ASD, as there is a greater prevalence and elevated risk of developing ASD in regions where pesticide exposure is high.

Neurodevelopmental disorders encompass a diverse range of conditions, including intellectual disability, specific learning disorders, communication disorders, motor disorders, attention-deficit/hyperactivity disorder (ADHD), and ASD [57]. Among these, ADHD and ASD have been the primary focus of studies investigating the potential impact of pesticide exposure during the prenatal and postnatal periods. Regarding this matter, the findings are inconclusive due to the variation in results observed across different studies. The inconsistency of the results could stem from differences in study designs, measurement tools, or the age range of the children involved [27,58]. For instance, while a positive correlation has been demonstrated between pyrethroid exposure and an elevated odds of developing ADHD and ADHD symptoms in children, other researchers have not observed a significant association [58]. In the present study, the results show an association between exposure to pesticides and autism. Our findings align with a previous study that discovered an association between prenatal exposure to various compounds such as glyphosate, chlorpyrifos, diazinon, malathion, ivermectin, and permethrin and the risk of developing ASD or lower performance on neurodevelopmental tests [27,59].

The multiple logistic regression analysis conducted to assess ASD with adjustments made for age, gender, and environmental exposure to pesticides uncovered a notable trend: individuals residing in regions characterized by substantial pesticide usage exhibited a heightened propensity to develop the condition. This finding is particularly significant in the context of gender stratification, where a nuanced observation emerged. Specifically, upon delving deeper into the data based on gender, it became evident that males inhabiting districts with elevated pesticide exposure levels faced a considerably greater risk of ASD manifestation compared to their female counterparts. Based on epidemiological data, it is estimated that the ratio of males to females diagnosed with autism is approximately 3:1 [60]. In relation to pesticides, males experience a greater deficit in working memory than females following prenatal exposure to chlorpyrifos [61]. 

This gender gap in autism prevalence has been extensively researched and remains incompletely understood. Previous studies conducted on animals have indicated that exposure to pesticides like chlorpyrifos elicits varying cognitive and emotional responses in males and females. Specifically, it has been observed that high doses of chlorpyrifos can lead to notable impairments in social behavior among male offspring of chlorpyrifos-treated mothers, in contrast to female offspring [62]. Additionally, separate research has shown that gestational exposure to chlorpyrifos is associated with heightened levels of anxiety, reduced intensity of aggressive behaviors during lactation in females, as well as delayed initiation of social investigation and a diminished response to social novelty [63,64]. In summary, it is evident that vulnerability to the effects of pesticides and the development of conditions resembling ASD may exhibit variation between males and females owing to inherent biological and hormonal disparities [65].

Multiple investigations have sought to unravel the mechanisms by which pesticides can inflict damage on the organism. Pesticides primarily result in disruptions to the endocrine system, immune system, and nervous system, as well as reproductive damage and an increased risk of cancer [66,67,68]. Hence, there is reason to suspect that both hormonal and neurotoxic effects of pesticides could interfere with the proper development of the fetus’s nervous system. Thus, the behavior of pesticides or commercial formulations based on pesticides as endocrine disruptors has been well established since they interfere with aromatase activity or estrogen induced-signaling, among others [66,67,69]. Similarly, glyphosate, a broad-spectrum herbicide used in various countries, promotes the proliferation of estrogen-dependent breast cancer cells by activating the estrogen receptor [70]. The possible effects of pesticide exposure during pregnancy have been investigated, revealing them as compounds capable of generating negative consequences for both the health of the mother and the development of the fetus. These effects encompass pregnancy complications, preterm birth, reduced birth weight, and the occurrence of congenital malformations [71,72,73,74].

The observed neurotoxic effects primarily consist of modifications in neurotransmitter and receptor levels, along with the disruption of their regulatory enzymes. Numerous studies conducted on experimental animals extensively document the impact of these effects on the nervous system [75,76,77]. Certain pesticides have the potential to influence the release, reuptake, metabolism, or receptors of neurotransmitters such as dopamine, glutamate, serotonin, or gamma-aminobutyric acid (GABA) [78]. In line with this, exposure to glyphosate-based herbicide during the prenatal and postnatal stages leads to neuronal damage through glutamate excitotoxicity and alteration of N-methyl-D-aspartate (NMDA) receptors in the hippocampus of immature and adult offspring [76,79]. Furthermore, another study revealed that exposure to various doses of glyphosate-based herbicide resulted in delayed innate reflexes, deficits in motor development, sociability, short and long-term learning, and memory. These effects were linked to changes in the cholinergic and dopaminergic systems [75]. Additionally, exposure of pregnant and lactating mouse mothers to deltamethrin, a pyrethroid, led to an increase in repetitive behaviors, disrupted fear conditioning and operant conditioning, and caused striatal dopaminergic dysfunction [77].

There is a suggestion that pesticide exposure could contribute to the development of autism by triggering neuroinflammation and impairing immune function. In laboratory experiments, it has been noted that the exposure of microglial cells to pyrethroid pesticides such as permethrin and deltamethrin triggers their activation, resulting in an elevated release of tumor necrosis factor α (TNF-α) [80]. Similarly, exposure to glyphosate-based herbicide resulted in a significant elevation in the expression levels of TNF-α mRNA in the hippocampus compared to the control group [81]. Excessive activation of microglia and the presence of chronic inflammation can lead to alterations in neuronal connections, synaptic plasticity, and other important neurobiological processes during cerebral development [82,83].

### 4.1. Limitations

The main limitation of this study is the reliance on ecological data for pesticide exposure. Due to the unavailability of individual-level information regarding the frequency and duration of pesticide exposure, we had to utilize aggregated exposure measures to assess the risk of developing ASD. While aggregated exposure data may not precisely reflect individual-level exposure, the classification of health districts into high and low pesticide use aimed to create relatively consistent exposure levels resembling the average exposure for individuals residing in areas with high and low pesticide exposure.

The potential bias arising from socioeconomic factors was mitigated as no significant differences were observed in the distribution of the working population across the primary, secondary, and tertiary sectors of economic activity in regions with high and low pesticide usage. While statistically significant differences were observed, these arose due to the primary sector predominating in the high pesticide exposure area, while the secondary sector exhibited a greater prevalence in the low pesticide exposure area. The tertiary sector, which holds considerable socioeconomic influence, displayed a comparable distribution across both study regions (Table 4). However, no disparities in personal disposable income between high and low pesticide exposure areas were noted. Overall, it can be inferred that socioeconomic factors do not appear to play a significant role in the impact on ASD risk in our study. Conversely, the key advantage of this study lies in its realistic examination of pesticide exposure within a large region characterized by high-intensity agriculture over a 21-year period.

### 4.2. Future Directions

Recognizing risk factors such as pesticide usage is crucial in both preventing and effectively managing ASD. The acknowledgment that pesticides pose a risk could serve as a catalyst for the implementation of strategies aimed at reducing exposure, including the reinforcement of stricter agricultural regulations, the promotion of sustainable farming practices, and advocacy for the adoption of less harmful pest control methods. Additionally, it is imperative to conduct further investigations to gain a deeper understanding of the underlying causes and associated risk factors of ASD, especially given the current limitations in available epidemiological evidence. Furthermore, continued exploration of potential risk factors and the adoption of a comprehensive approach to ASD prevention, which encompasses the promotion of healthy lifestyles and the provision of lifelong support for cognitive and motor health, are essential.

## 5. Conclusions

The prevalence of ASD was significantly higher in regions with extensive pesticide use, particularly among males, suggesting a potential link between pesticide exposure and autism. These findings underscore the importance of further research and awareness regarding the potential connection between pesticide exposure and ASD.

## Figures and Tables

**Table 1:** Comparison of the ASD individuals’ mean ages by the geographical areas studied (high and low use pesticide) and sex.

ASD	Exposure	Age (Mean (SD))	*p* Value *
Males	High pesticide use	5.08 (3.33)	0.06 *
	Low pesticide use	5.25 (3.04)	
Females	High pesticide use	4.89 (3.38)	0.43 *
	Low pesticide use	4.93 (3.08)	
Total	High pesticide use	4.96 (3.34)	0.06 *
	Low pesticide use	5.15 (3.05)	

* *p*-value obtained by Mann–Whitney U test.

**Table 2:** Prevalence (rate per 100 inhabitants), odds ratio (OR), and 95% confidence interval (95% CI) for ASD in the population living in areas of high pesticide use relative to areas of low pesticide use.

ASD	High Pesticide Use	Low Pesticide Use	OR (95% CI)	*p* Value *
Total	1.03	0.76	1.34 (1.24–1.44)	<0.001
Males	1.47	1.03	1.42 (1.30–1.55)	<0.001
Females	0.56	0.48	1.17 (1.10–1.34)	0.02

* *p*-value obtained by Pearson’s Chi-squared test.

**Table 3:** Stepwise multiple logistic regression analysis of ASD, adjusted for the geographical areas studied (high and low pesticide exposure) and gender.

	Risk Factor	OR *	OR (95% CI)	*p* Value *
ASD	Areas of high pesticide use	1.52	1.41–1.64	<0.001
	Male	2.41	2.21–2.62	<0.001

* Models were adjusted for the following variables: age, sex (1: male, 0: female), environmental pesticide exposure (1: areas of high pesticide use, 0: areas of low pesticide use).

**Table 4:** Distribution of employed population by economic activity and average disposable personal incomes.

	High Pesticide Use	Low Pesticide Use	*p* Value
Economic sectors *			
Primary sector (agriculture and livestock, forestry and fishing)	56,658 (30.53%)	9845 (21.79%)	<0.001
Secondary sector (manufacturing industry and construction)	32,678 (17.60%)	13,462 (29.79%)	
Tertiary sector (service industries, private and public activities)	96,231 (51.85%)	21,870 (48.40%)	
Disposable personal income ** (Euros/person)	13,567 ± 1.77	14,120 ± 1.20	0.059

* Data obtained from the 2021 database from the Institute of Statistic of Andalusia. ** Source: Spanish Tax Agency [accessed on 28 February 2024] Available at: https://sede.agenciatributaria.gob.es/AEAT/Contenidos_Comunes/La_Agencia_Tributaria/Estadisticas/Publicaciones/sites/irpf/2021/home_parcial2864d0b295b467acb2ef7439b8d50298efe39e5f.html.

## Data Availability

The data that support the findings of this study are available on request from the corresponding author.
